# Perceived information provision and satisfaction among lymphoma and multiple myeloma survivors—results from a Dutch population-based study

**DOI:** 10.1007/s00277-012-1495-1

**Published:** 2012-05-26

**Authors:** Simone Oerlemans, Olga Husson, Floortje Mols, Philip Poortmans, Henk Roerdink, Laurien A. Daniels, Carien L. Creutzberg, Lonneke V. van de Poll-Franse

**Affiliations:** 1 Research Department, Comprehensive Cancer Centre South, PO Box 231, 5600 AE Eindhoven, the Netherlands; 2 Center of Research on Psychology in Somatic Diseases (CoRPS), Tilburg University, Tilburg, the Netherlands; 3 Department of Radiation therapy, Institute Verbeeten, Tilburg, the Netherlands; 4 Department of Oncology and Hematology, Twee Steden Hospital, Tilburg, the Netherlands; 5 Department of Clinical Oncology, Leiden University Medical Center, Leiden, the Netherlands

**Keywords:** Information provision, Patient satisfaction, Lymphoma, Multiple myeloma, Cancer survivors, Population-based

## Abstract

To improve posttreatment care for (long-term) lymphoma survivors in the Netherlands, survivorship clinics are being developed. As information provision is an important aspect of survivorship care, our aim was to evaluate the current perceived level of and satisfaction with information received by non-Hodgkin’s lymphoma (NHL), Hodgkin’s lymphoma (HL) and multiple myeloma (MM) survivors, and to identify associations with sociodemographic and clinical characteristics. The population-based Eindhoven Cancer Registry was used to select all patients diagnosed with NHL, HL and MM from 1999 to 2009. In total, 1,448 survivors received a questionnaire, and 1,135 of them responded (78.4 %). The EORTC QLQ-INFO25 was used to evaluate the perceived level of and satisfaction with information. Two thirds of survivors were satisfied with the amount of received information, with HL survivors being most satisfied (74 %). At least 25 % of survivors wanted more information. Young age, having had chemotherapy, having been diagnosed more recently, using internet for information and having no comorbidities were the most important factors associated with higher perceived levels of information provision. Although information provision and satisfaction with information seems relatively good in lymphoma and MM survivors, one third expressed unmet needs. Furthermore, variations between subgroups were observed. Good information provision is known to be associated with better quality of life. Survivorship care plans could be a way to achieve this.

## Introduction

On January 1, 2009 there were approximately 21,000 non-Hodgkin lymphoma (NHL), 5,300 Hodgkin lymphoma (HL), and 3,300 multiple myeloma (MM) survivors in the Netherlands [1]. These numbers are expected to increase to approximately 32,000 NHL, 6,300 HL and 4,300 MM survivors by 2020 [1]. This substantial rise will result in an increasing health care burden in haematology, especially indolent lymphomas and MM, which both are characterised by a prolonged clinical course with repeated relapses and slow but ongoing progression [2].

To improve care for this growing group of cancer survivors, a nationwide initiative of haematologists, radiation oncologists, epidemiologists and internists has founded a Working Group named “BETTER” (“BETER” in Dutch), which is currently developing protocols for standardized long-term care for HL and NHL survivors and establishing survivorship clinics. The goals of these clinics are to minimize the occurrence and influence of late effects and to improve survivors’ quality of life (QoL) by: informing survivors about long-term risks, advice preventive measures, suggest screening and improve aftercare by providing rehabilitation programmes [3].

Patient information is an essential component of cancer care and rehabilitation [4]. Patients, who are well informed about their cancer, treatment, and aftercare, are more likely to complete their therapy and are less anxious thereafter [5, 6]. Providing adequate information to cancer patients can reduce the psychological burden and improve patients’ QoL and their satisfaction with care [7, 8]. This is important since lymphoma and MM survivors report lower QoL compared to normative populations even years after diagnosis [9, 10].

Up to now, no studies have investigated the perceived level of and satisfaction with information provision in NHL, HL and MM survivors. If factors associated with information satisfaction are known, health care providers can better give adequate information to those who need it, which can contribute to an improved quality of care and QoL. The aim of the present study was therefore to measure the perceived level of, and satisfaction with information received by survivors of indolent NHL (I-NHL), aggressive NHL (A-NHL), HL and MM, and to identify associations with sociodemographic and clinical characteristics for each tumour type.

## Methods

### Setting and population

This study is part of a dynamic longitudinal population-based survey among lymphoma and MM survivors registered within the Eindhoven Cancer Registry (ECR) of the Comprehensive Cancer Centre South and is embedded in Population-Based HAematological Registry for Observational Studies. The ECR records data on all patients who are newly diagnosed with cancer in the southern part of the Netherlands, an area with 2.3 million inhabitants, 18 hospital locations and 2 large radiotherapy institutes. The ECR was used to select all patients who were diagnosed with NHL, HL and MM between January 1, 1999 and January 1, 2009. We included all subtypes of indolent (including chronic lymphocytic leukaemia-like) and aggressive B cell NHL, HL and MM as defined by the International Classification of Diseases for Oncology-3 codes [11].

Deceased patients were excluded by linking the ECR database with the Central Bureau for Genealogy. Ethical approval for the study was obtained from a regional, certified Medical Ethics Committee.

### Data collection

Data collection took place in 2009 and was done within PROFILES (Patient Reported Outcomes Following Initial treatment and Long-term Evaluation of Survivorship). PROFILES is a registry for the study of the physical and psychosocial impact of cancer and its treatment from a dynamic, growing population-based cohort of both short- and long-term cancer survivors. PROFILES contains a large web-based component and is linked directly to clinical data from ECR. Details of the data collection method have been previously described [12]. Data from the PROFILES registry will become available for noncommercial scientific research, subject to study question, privacy and confidentiality restrictions, and registration (www.profilesregistry.nl).

In May 2009, patients between 1 and 10 years after diagnosis were included in the study and received the first questionnaire. In November 2009, patients diagnosed between May and November 2009 were invited to participate.

### Study measures

The Dutch version of the European Organisation for Research and Treatment of Cancer (EORTC) QLQ-INFO25 questionnaire was used to evaluate the perceived level of and satisfaction with information among NHL, HL and MM patients [13]. This 25-item questionnaire incorporates four information provision subscales: perceived receipt of information about the disease, medical tests, treatment and other care services. Additionally, it contains several single items on receiving written information or information on CD or tape/video and items on the satisfaction with and helpfulness of the received information. Answer categories range from one (not at all) to four (very much), except for four items which have a two-point scale. Furthermore, an open question is asked on what topics survivors would like to receive more information on. After linear transformation, all scales and items range in scores from 0 to 100, with higher scores indicating better perceived information provision. The questionnaire has been internationally validated, and internal consistency for all scales is good (*ɑ* > 0.70), as is test–retest reliability (interclass correlations >0.70) [14]. Our data revealed Cronbach’s alphas of 0.75 (disease), 0.88 (medical test), 0.88 (treatment) and 0.82 (other services) for the four subscales, respectively. In addition to the EORTC QLQ-INFO25, we asked patients two single questions about the use of internet for seeking additional information, which could be answered with either yes or no.

Comorbidity at time of survey was categorized according to the adapted Self-administered Comorbidity Questionnaire [15]. Questions on survivors’ marital status and educational level were also added to the questionnaire. Clinical information was available from the ECR that routinely collects data on tumour characteristics, including date of diagnosis, histology, Ann Arbor stage (where appropriate) [16], primary treatment and patients’ background characteristics, including gender and date of birth.

### Statistical analysis

All statistical analyses were performed using SPSS version 17.0 (Statistical Package for Social Sciences, Chicago, IL, USA) and *p* values of <.05 were considered statistically significant. For the EORTC QLQ-INFO25, we used a score of ≥10 points difference on subscales to define a clinical important difference [17].

Differences in sociodemographic and clinical characteristics between respondents, non-respondents and patients with unverifiable addresses and between tumour types were compared with a chi-square, *t* test or its nonparametric equivalent where appropriate.

Multi-item scales of the EORTC QLQ-INFO25 were included in the analyses if at least half of the items from the scale were answered, according to the EORTC QoL guidelines [13, 14, 18]. ANOVA and chi-square were performed to investigate mean differences between tumour type (independent variables) and the EORTC QLQ-INFO25 scales (dependent variables).

Multivariate regression analyses were performed to investigate the independent association of sociodemographic and clinical characteristics with the subscales of the EORTC QLQ-INFO25. All sociodemographic and clinical variables were included; this was determined a priori. Stage was only included in the analyses for A-NHL and HL since it was not available for I-NHL and MM (Table 1).

**Table 1:** Sociodemographic and clinical characteristics of questionnaire respondents, non-respondents and patients with unverifiable addresses

	Respondents	Non-respondents	Patients with unverifiable addresses	*p* value
	*N* = 1,135	*N* = 313	*N* = 271	
Tumour type				0.06
I-NHL	443 (39 %)	140 (45 %)	110 (41 %)	
A-NHL	375 (33 %)	80 (26 %)	82 (30 %)	
HL	164 (14 %)	37 (12 %)	44 (16 %)	
MM	153 (14 %)	56 (23 %)	35 (13 %)	
Age (at time of survey) (mean ± SD)	61.6 (14)	60.5 (16)	57.2 (16)	<0.01
<55	312 (28 %)	104 (33 %)	113 (42 %)	
55–69	452 (40 %)	99 (32 %)	79 (29 %)	
≥70	369 (33 %)	110 (35 %)	79 (29 %)	
Years since diagnosis (mean ± SD)	3.7 (2.7)	3.2 (3.0)	3.9 (2.9)	<0.01
0–1	313 (28 %)	130 (42 %)	71 (26 %)	
2–4	422 (37 %)	92 (29 %)	102 (38 %)	
5–7	264 (23 %)	46 (15 %)	56 (21 %)	
8–10	136 (12 %)	45 (14 %)	42 (16 %)	
Gender				0.38
Male	677 (60 %)	184 (59 %)	147 (55 %)	
Female	457 (40 %)	127 (41 %)	120 (45 %)	
Stage at diagnosis				<0.01
I	248 (22 %)	52 (17 %)	65 (24 %)	
II	220 (19 %)	57 (18 %)	39 (14 %)	
III	183 (16 %)	40 (13 %)	42 (16 %)	
IV	218 (19 %)	50 (16 %)	58 (21 %)	
Unknown	266 (23 %)	114 (36 %)	67 (25 %)	
Primary treatment				
Radiotherapy	88 (7.8 %)	17 (5.4 %)	20 (7.4 %)	0.09
Chemotherapy	515 (45 %)	118 (38 %)	106 (39 %)	0.02
Chemotherapy + radiotherapy	239 (21 %)	56 (18 %)	52 (19 %)	0.11
Active surveillance^a^	233 (21 %)	89 (23 %)	71 (26 %)	<0.01
Stem cell transplantation	58 (5.1 %)	16 (5.1 %)	8 (3.0 %)	0.07

*I-NHL* indolent non-Hodgkin lymphoma, *A-NHL* aggressive non-Hodgkin lymphoma, *HL* Hodgkin lymphoma, *MM* multiple myeloma

^a^Patients are under active surveillance and receive no therapy

Logistic regression analyses were performed with received information satisfaction as outcome measure, one for the total group and four for the tumour types. Therefore, patients were categorized into two groups: (a) patients who were unsatisfied or only a little satisfied, classified as unsatisfied and (b) patients who were quite satisfied or very satisfied, classified as satisfied. Again, all sociodemographic and clinical variables were included. Stage was only included in the analyses for A-NHL and HL since stage was not available in I-NHL and MM.

## Results

### Patient and tumour characteristics

Of the 1,448 lymphoma and MM survivors who were sent a questionnaire, 1,135 (78 %) completed it. Non-respondents were more recently diagnosed and less often diagnosed with stage I disease. Furthermore, they were less often treated with chemotherapy compared to respondents. Patients with unverifiable addresses were younger, diagnosed less recent, less often treated with chemotherapy and more often had active surveillance as primary treatment compared to respondents. There were no differences in response according to tumour type or gender (Table 1).

Participating HL survivors were significantly younger, more often had a job and reported fewer comorbid conditions than I-NHL, A-NHL and MM survivors. MM survivors were most recently diagnosed compared to the other three tumour groups (Table 2).

**Table 2:** Sociodemographic and clinical characteristics of cancer survivors, stratified by tumour type

	I-NHL	A-NHL	HL	MM	*p* value
	*N* = 443	*N* = 375	*N* = 164	*N* = 153	
Age (at time of survey) (mean ± SD)	64.1 (11)	63.3 (14)	46.6 (15)	66.1 (10)	<0.01
<55	90 (20 %)	90 (24 %)	112 (69 %)	20 (13 %)	
55–69	199 (45 %)	136 (36 %)	38 (23 %)	79 (52 %)	
≥70	154 (35 %)	148 (40 %)	13 (8.0 %)	54 (35 %)	
Years since diagnosis (mean ± SD)	4.0 (2.7)	3.5 (2.6)	4.4 (2.9)	2.4 (2.3)	<0.01
0–1	100 (23 %)	108 (29 %)	36 (22 %)	69 (45 %)	
2–4	169 (38 %)	144 (38 %)	50 (31 %)	59 (39 %)	
5–7	113 (26 %)	85 (23 %)	49 (30 %)	17 (11 %)	
8–10	61 (14 %)	38 (10 %)	29 (18 %)	8 (5.2 %)	
Gender					0.10
Male	266 (60 %)	239 (64 %)	89 (54 %)	83 (55 %)	
Female	177 (40 %)	136 (36 %)	75 (46 %)	69 (45 %)	
Stage at diagnosis					<0.01
I	NA	118 (32 %)	30 (18 %)	NA	
II	NA	90 (24 %)	83 (51 %)	NA	
III	NA	68 (18 %)	33 (20 %)	NA	
IV	NA	93 (25 %)	17 (10 %)	NA	
Unknown	NA	6 (1.6 %)	1 (0.6 %)	NA	
Primary treatment					
Radiotherapy (only)	64 (14 %)	12 (3.2 %)	4 (2.4 %)	8 (5.2 %)	<0.01
Chemotherapy (only)	157 (35 %)	235 (63 %)	65 (40 %)	58 (38 %)	<0.01
Chemotherapy + radiotherapy	14 (3.2 %)	98 (26 %)	94 (57 %)	33 (22 %)	<0.01
Active surveillance^a^	187 (42 %)	25 (6.7 %)	1 (0.6 %)	20 (13 %)	<0.01
Stem cell transplantation	8 (1.8 %)	22 (5.9 %)	0 (0 %)	28 (18 %)	<0.01
Comorbidity					<0.01
None	108 (26 %)	103 (30 %)	75 (48 %)	26 (19 %)	
1	122 (30 %)	118 (34 %)	46 (30 %)	43 (31 %)	
2	90 (22 %)	65 (19 %)	14 (9.0 %)	35 (26 %)	
3 or more	90 (22 %)	60 (17 %)	20 (13 %)	33 (24 %)	
Marital status					0.41
Partner	353 (81 %)	287 (79 %)	122 (75 %)	116 (77 %)	
No partner	84 (19 %)	77 (21 %)	41 (25 %)	35 (23 %)	
Education level					0.11
Low	69 (16 %)	62 (17 %)	16 (9.8 %)	30 (20 %)	
Medium	264 (61 %)	219 (61 %)	99 (61 %)	95 (63 %)	
High	101 (23 %)	80 (22 %)	48 (29 %)	27 (18 %)	
Current occupation					<0.01
Employed	166 (46 %)	128 (45 %)	112 (84 %)	39 (34 %)	
Not working/retired	198 (54 %)	155 (55 %)	21 (16 %)	76 (66 %)	
Follow-up care					<0.01
No	42 (10 %)	32 (10 %)	12 (8 %)	30 (24 %)	
2–4 times a year	324 (80 %)	245 (74 %)	81 (52 %)	95 (75 %)	
Once a year	35 (9 %)	52 (16 %)	62 (40 %)	1 (1 %)	
Once every 2 years	3 (1 %)	2 (1 %)	1 (1 %)	0 (0 %)	

*I-NHL* indolent non-Hodgkin lymphoma, *A-NHL* aggressive non-Hodgkin lymphoma, *HL* Hodgkin lymphoma, *MM* multiple myeloma, *NA* not available; education levels included *low* no/primary school, *medium* lower general secondary education/vocational training, or *high* pre-university education/high vocational training/university.

^a^Patients are under active surveillance and receive no therapy

### Satisfaction with and amount of information

Satisfied cancer survivors (*n* = 724; 67 %) perceived to have received more information (disease, medical tests, treatment and other services) and found the information more useful than dissatisfied patients (*n* = 411; 33 %), with mean differences ranging between 46 and 74 points (all *p* < 0.01).

In total, 29 % of survivors would have liked to receive more information (29 % I-NHL, 25 % A-NHL, 30 % HL and 29 % MM). Most frequently mentioned topics to receive more information about were cause and course of disease (45 % I-NHL, 59 % A-NHL, 24 % HL and 54 % MM), late effects of treatment (46 % I-NHL, 37 % A-NHL, 50 % HL and 30 % MM) and psychosocial aftercare (10 % I-NHL, 23 % A-NHL, 26 % HL and 30 % MM).

### Associations with perceived level of and satisfaction with information

Mean scores on perceived level of and satisfaction with information on all scales were the highest for HL survivors and the lowest for I-NHL survivors (Table 3). Furthermore, HL survivors found the information more useful compared to all other tumour groups.

**Table 3:** Mean EORTC QLQ-INFO25 subscale scores (±SD) according to tumour type

	I-NHL	A-NHL	HL	MM	*p* value
	*N* = 443	*N* = 375	*N* = 164	*N* = 153	
EORTC QLQ-INFO25	Mean (SD)	Mean (SD)	Mean (SD)	Mean (SD)	
Information about disease	50 (22)	53 (20)	56 (16)	51 (22)	<0.05^a^
Information about medical tests	63 (22)	64 (23)	68 (21)	65 (23)	0.15
Information about treatment	41 (24)	50 (21)	57 (19)	47 (24)	<0.01^b^
Information about other services	16 (21)	25 (24)	27 (22)	22 (21)	<0.01^c^
Satisfaction with information	60 (28)	61 (26)	66 (25)	61 (28)	0.15
Usefulness of information	62 (25)	66 (24)	73 (21)	62 (25)	<0.01^d^
	% Yes	% Yes	% Yes	% Yes	
Want more information	29 %	25 %	30 %	29 %	0.48
Want less information	3 %	3 %	2 %	1 %	0.74

EORTC-QLQ INFO25 scales 0–100: a higher score reflects better perceived information received

*I-NHL* indolent non-Hodgkin lymphoma, *A-NHL* aggressive non-Hodgkin lymphoma, *HL* Hodgkin lymphoma, *MM* multiple myeloma

^a^Between I-NHL and HL

^b^Between I-NHL and A-NHL, HL, MM; between HL and A-NHL, MM

^c^Between I-NHL and A-NHL, HL, MM

^d^Between HL and I-NHL, A-NHL, MM

Multivariate linear regression analyses including all patients in one model showed that receiving more disease-related information was associated with having no comorbid conditions, using internet for information and hospital (*β* = .11; *p* < .01; Table 4). More information on medical tests was associated with less comorbidity, high education and use of internet. Furthermore, receiving more information about treatment and other services was associated with younger age, having had chemotherapy, less comorbidity and hospital (*β* between .08 and .10; *p* < .05). Being diagnosed with I-NHL and being under active surveillance were associated with a lower perceived level of receiving information about treatment. Satisfaction with information was independently associated with having had chemotherapy and negatively associated with comorbidity.

**Table 4:** Standardized betas of multivariate linear regression analyses evaluating the association of independent variables with the information provision subscales

	Disease	Medical tests	Treatment	Other	Satisfaction with received information
	(Beta)	(Beta)	(Beta)	(Beta)	(odds ± 95 % CI)
Tumour type					
I-NHL	−0.07	−0.07	−0.12**	−0.09	0.89 (0.52–1.52)
A-NHL	−0.05	−0.07	−0.04	0.03	0.783 (0.48–1.28)
HL	Ref	Ref	Ref	Ref	Ref
MM	−0.05	0.02	−0.03	0.00	0.81 (0.43–1.56)
Age	−0.05	0.01	−0.12**	−0.11**	1.00 (0.99–1.01)
Years since diagnosis	−0.01	0.01	0.02	−0.05	0.97 (0.92–1.03)
Gender					
Male	Ref	Ref	Ref	Ref	Ref
Female	0.01	0.02	−0.01	−0.01	0.77 (0.58–1.03)
Chemotherapy					
No	Ref	Ref	Ref	Ref	Ref
Yes	0.03	−0.01	0.14*	0.14**	1.81 (1.04–3.13)*
Radiotherapy					
No	Ref	Ref	Ref	Ref	Ref
Yes	−0.08	−0.06	−0.06	−0.07	1.00 (0.68–1.45)
Active surveillance					
No	Ref	Ref	Ref	Ref	Ref
Yes	−0.09	−0.08	−0.16**	−0.06	1.39 (0.76–2.55)
Stem cell transplantation					
No	Ref	Ref	Ref	Ref	Ref
Yes	0.07	0.06	0.05	0.06	1.51 (0.73–3.13)
Comorbidity					
None	Ref	Ref	Ref	Ref	Ref
1	−0.07	−0.04	−0.07	−0.02	0.74 (0.51–1.52)
2	−0.07	−0.05	−0.14**	−0.03	0.55 (0.36–0.85)**
3 or more	−0.90*	−0.90*	−0.07*	−0.01	0.55 (0.36–0.84)**
Marital status					
Partner	Ref	Ref	Ref	Ref	Ref
No partner	0.01	0.02	−0.02	0.00	1.21 (0.84–1.73)
Education level					
Low	0.02	−0.04	−0.02	−0.03	0.85 (0.52–1.38)
Medium	−0.03	−0.08*	−0.06	−0.05	0.81 (0.57–1.16)
High	Ref	Ref	Ref	Ref	Ref
Use of internet					
Yes	Ref	Ref	Ref	Ref	Ref
No	−0.08*	−0.07*	−0.04	−0.03	0.97 (0.71–1.32)

*I-NHL* indolent non-Hodgkin lymphoma, *A-NHL* aggressive non-Hodgkin lymphoma, *HL* Hodgkin lymphoma, *MM* multiple myeloma; Education levels included *low* no/primary school, *medium* lower general secondary education/vocational training, or *high* pre-university education/high vocational training/university

*p < .05; **p < .01

Additional multivariate analyses within the different tumour types showed similar findings (data not shown in table). Younger age (*β* between −.13 and −.46; *p* < .05) and a more recent diagnosis (*β* between −.10 and −.20; *p* < .05) were frequently positively associated with perceived information provision, whereas comorbidity (*β* between −.13 and −.23; *p* < .05) was frequently negatively associated with perceived information provision.

I-NHL survivors with a low or medium educational level reported lower levels of treatment information (*β* = −.15; *p* < .05) compared to those who were highly educated. A-NHL survivors with stage II or III disease (*β* = .22; *p* < .01) or those who received chemotherapy (*β* = .17; *p* < .01) reported higher perceived levels of information compared to those who did not. HL survivors with a low educational level (*β* = .23; *p* < .05) and those using internet (*β* = −.18; *p* < .05) reported higher levels of perceived information. Lastly, MM survivors under active surveillance reported lower perceived levels of information about treatment (*β* = −.45; *p* < .05) compared to patients who were actively treated.

## Discussion

In the present study among 1,135 NHL, HL and MM survivors, two thirds of survivors were satisfied with the amount of received information about their haematological malignancy, respectively 65 % of I-NHL, 67 % of A-NHL, 74 % of HL and 68 % of MM survivors. However, variations were observed, and at least a quarter of survivors wanted more information, with large differences between hospitals (range, 24–40 %).

Younger age, having had chemotherapy, using internet for information and having no comorbid conditions appeared to be the most important factors associated with higher perceived levels of information provision. Analyses per tumour type showed similar findings. Worth mentioning is that in the analyses per tumour, I-NHL, A-NHL and MM survivors who had been diagnosed more recently had higher perceived levels of information provision, which possibly indicates that information provision has improved with time. However, it is also possible that recall bias influenced these findings, for those diagnosed more recently, the information received is still fresh in their memory and by the more frequent contacts with their physician in the phase more closely to diagnosis.

Our findings that the perceived level of information provision is associated with age, education, time since diagnosis and disease stage are in line with other studies [19–24]. Studies have shown that older and lower educated patients tend to ask fewer questions during their visit with their physician, and might therefore receive less information [25, 26]. Furthermore, older patients have been found to take a more passive role in interaction with their physician and have a greater reliance that their physician will provide all information [24]. In addition, higher educated patients are more likely to seek information from other sources such as the internet and consequently obtain more information [24].

The results of our study, with 67 % of survivors being satisfied with the amount of information received, were different compared to a study among mostly early-stage melanoma survivors in which only 39 % of survivors indicated to be satisfied [22]. These differences might be explained by the more chronic level and intense treatment of lymphoma and MM compared to early-stage melanoma. In addition, lymphoma and MM survivors will have more visits with the physician and therefore a possible improved information provision. Patients’ satisfaction is also influenced by patients’ expectations of the course of their disease [27]. Patients’ expectations can vary widely, depending of the type of tumour [27]. HL survivors may be more satisfied with and score better on perceived information since they have a better prognosis compared to NHL and MM survivors.

Survivors who were satisfied with the received information scored significantly and clinically relevant higher on all information provision subscales and on the usefulness of information scale compared to the unsatisfied survivors. To improve information provision in the group of unsatisfied survivors, physicians could screen their patients by asking if they are satisfied with the amount of information received, and when unsatisfied, physicians can ask what the patients’ information needs are.

To provide the needed (written) information to patients, physicians should think of the educational level of the information provision. Patients with a lower educational level and patients with a low level of literacy will need extra help to understand the information. In the USA, more attention is being paid to health literacy [20, 21, 28], i.e. “the degree to which individuals have the capacity to obtain, process and understand basic health information and services needed to make appropriate health decisions” [29], than in the Netherlands. Since our and other studies have observed that lower educated survivors report worse scores, more attention should be paid to providing information on a basic comprehensive level [19, 22, 23].

One third of survivors would have liked to receive more information. The topic that was mentioned most often was information on late effects (37–50 %) followed by information on the cause and course of the disease (24–59 %) and psychosocial aftercare (10–26 %). Inviting survivors for the “BETTER” initiative could be an efficient solution to address these lasting information needs and leads to improved health care perception.

The present study has a few limitations. Although information was present concerning demographic and clinical characteristics of the non-respondents and patients with unverifiable addresses, it remains unknown why non-respondents declined to participate in the study. In addition, the cross-sectional design of our study limits the determination of causal associations between the study variables. Furthermore, the mean time since diagnosis was 3.7 years, which could influence the recall effect of information received. However, in the case of indolent lymphoma and MM patients who visit their physicians more often, this may not have been a major problem as the majority of those patients (95 %) was still under active follow-up.

The strengths of our study are the population-based sampling frame instead of a hospital-based sampling frame, the high response rate and the large range in elapsed time since diagnosis. This facilitates to extrapolate the results to a broad range of lymphoma and MM survivors.

In conclusion, although information provision and satisfaction with information is relatively good in lymphoma and MM survivors, one third of the survivors were not satisfied with the perceived information provision, and variations between subgroups of patients were observed. The differences found between the participating hospitals with an assumed similar patient population suggest that there remains room for improvement. As survival of NHL, HL and MM has improved over the past decades and the numbers of long-term survivors increase, late effects of therapy become more important. Optimal, tailor-made and repeated information provision will lead to improved patient satisfaction and QoL. Implementation of survivorship care plans could contribute to the improvement of information provision [30].
